# New Insights into the Structure of (1→3,1→6)-β-D-Glucan Side Chains in the *Candida glabrata* Cell Wall

**DOI:** 10.1371/journal.pone.0027614

**Published:** 2011-11-11

**Authors:** Douglas W. Lowman, Lara J. West, Daniel W. Bearden, Michael F. Wempe, Trevor D. Power, Harry E. Ensley, Ken Haynes, David L. Williams, Michael D. Kruppa

**Affiliations:** 1 Department of Surgery, Quillen College of Medicine, East Tennessee State University, Johnson City, Tennessee, United States of America; 2 AppRidge International, LLC, Jonesborough, Tennessee, United States of America; 3 Department of Medicine, Imperial College London, London, United Kingdom; 4 Hollings Marine Laboratory, Analytical Chemistry Division, National Institutes of Standards and Technology, Charleston, South Carolina, United States of America; 5 School of Pharmacy, University of Colorado Health Sciences Center, Denver, Colorado, United States of America; 6 Department of Biochemistry and Molecular Biology, Sealy Center for Structural Biology and Molecular Biophysics, University of Texas Medical Branch, Galveston, Texas, United States of America; 7 Department of Chemistry, Tulane University, New Orleans, Louisiana, United States of America; 8 Department of Microbiology, Quillen College of Medicine, East Tennessee State University, Johnson City, Tennessee, United States of America; Louisiana State University, United States of America

## Abstract

β-glucan is a (1→3)-β-linked glucose polymer with (1→6)-β-linked side chains and a major component of fungal cell walls. β-glucans provide structural integrity to the fungal cell wall. The nature of the (1–6)-β-linked side chain structure of fungal (1→3,1→6)-β-D-glucans has been very difficult to elucidate. Herein, we report the first detailed structural characterization of the (1→6)-β-linked side chains of *Candida glabrata* using high-field NMR. The (1→6)-β-linked side chains have an average length of 4 to 5 repeat units spaced every 21 repeat units along the (1→3)-linked polymer backbone. Computer modeling suggests that the side chains have a bent curve structure that allows for a flexible interconnection with parallel (1→3)-β-D-glucan polymers, and/or as a point of attachment for proteins. Based on these observations we propose new approaches to how (1→6)-β-linked side chains interconnect with neighboring glucan polymers in a manner that maximizes fungal cell wall strength, while also allowing for flexibility, or plasticity.

## Introduction

Fungal cells are surrounded by a wall that consists of four main components: glucan, chitin, mannan and mannoprotein. These carbohydrates play an important role in maintaining the shape and viability of fungal cells in response to osmotic challenge, and they are the point of contact with the environment. A major carbohydrate component of the cell wall is (1→3)-β-D-glucan [1]. Glucans are generally arrayed as triple helical polymers, which function to maintain the rigidity of the cell wall [1]. The (1→3)-β-D-glucan is thought to form a lattice support structure through which the other molecules are attached. Chitin has been suggested to crosslink with both (1→3)- and (1→6)-β-D-glucan polymers [2]–[4] but the nature of the crosslink has not clearly been established. (1→6)-β-D-glucan side chain branches, attached to a (1→3)-β-D-glucan polymer backbone, have been reported for several different fungal species [5]–[8]. It is thought that the (1→6)-β-D-glucan serves as attachment sites for chitin, mannan, and mannoprotein [2], [3] and they may also serve to link or interconnect adjacent (1→3)-β-D-glucan polymer strands. However, the nature of the attachment to these carbohydrates has not been structurally characterized except that mannoprotein may be linked through a glycosylphosphatidylinositol anchor [9], [10]. Since (1→6)-β-D-glucan appears to serve a major role for attachment of these polymers, it is important to understand the structural nature of (1→6)-β-D-glucan as it exists when attached to the (1→3)-β-D-glucan polymer.

Previously, Tada and coworkers [8] characterized the structure of Grifolan-LE, isolated from the mushroom *Grifola frondosa*. This is a (1→3,1→6)-β-D-glucan polymer containing a (1→3)-β-linked backbone chain and side chains composed of a single (1→6)-β-D-glucan repeat unit attached, on average, to the backbone chain every 3 repeat units. In this current study, we have isolated glucan from wild-type and several mutant *C. glabrata* strains. Using a modified glucan extraction procedure, we have isolated a (1→3,1→6)-β-D-glucan that contains a unique poly-(1→6)-β-linked side chain structure. Based on extensive ^1^H and ^13^C 1D and homonuclear (COSY, NOESY and TOCSY) and heteronuclear (HSQC, HSQC-NOESY, HSQC-TOCSY, and HMBC) 2D NMR studies, we have determined that glucan isolated from *C. glabrata* contains a (1→3)-β-D-glucan polymer backbone with (1→6)-β-D-glucan-containing side chains composed of multiple (1→6)-β-linked glucosyl repeat units. Additionally, we have extended our investigation to include molecular modeling of the side chain structural conformation. The structure of the side chain appears to have a hook-like or curved structure, which may allow for interconnection with parallel (1→3)-β-D-glucan polymers, or serve as a point of attachment to chitin, mannan or a glycosylphosphatidylinositol anchor to a protein. Another possibility is that this hook-like structure plays a different role in the fungal cell wall structure, one that allows for physical grabbing of another (1→3)-β-D-glucan polymer, much in the same manner that a Velcro hook grabs a fiber strand. Based on these results, we propose that the structure of the (1→6)-β-D-glucan side chain may help to explain how the cell wall architecture allows for a rigid cell wall that also exhibits flexibility as the fungal cell grows.

## Results

### Elucidation of the (1→6)-β-linked side chain structure

**Figure 1:**
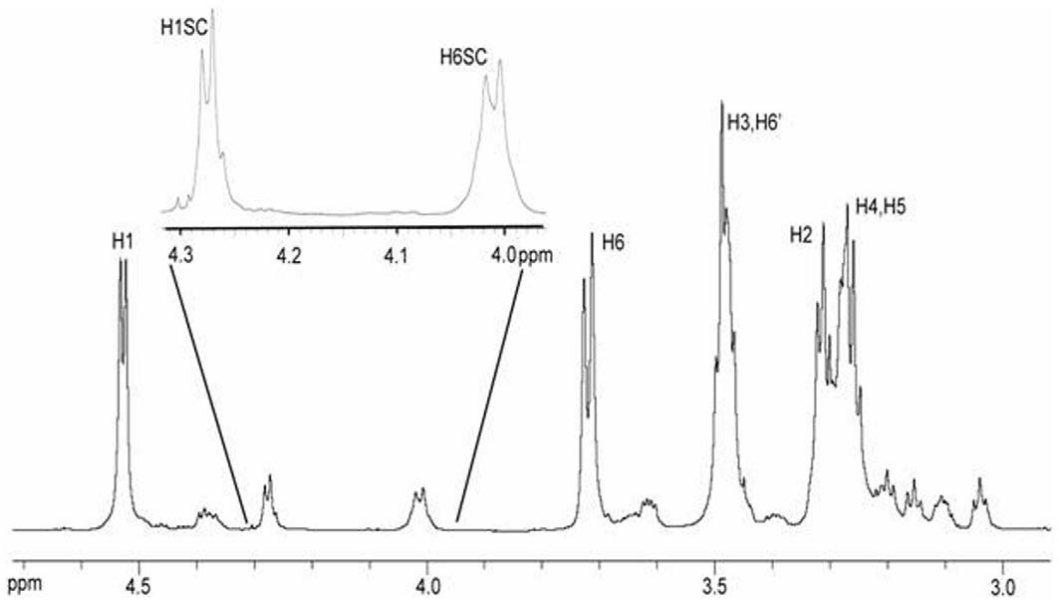
1D proton NMR spectrum of the (1→3,1→6)-β-D-glucan isolated from *C. glabrata ace2* strain. Proton assignments refer to the glucosyl repeat units in the polymer (1→3)-β-linked backbone. The expansion of the spectral region from 4.32 to 3.96 ppm shows the multiple inflection points in the resonances for the anomeric proton, H1SC, and one of the methylene protons, H6SC, of the (1→6)-β-linkage of the side chain. The resonance line shapes are suggestive for multiple side chain glycosidic bond conformations and/or multiple side chain lengths. The doublet resonance at 4.30 ppm results from the anomeric proton of β-glucose.

Tada and coworkers [8] recently presented the complete structural characterization of Grifolan-LE isolated from *G. frondosa*. Grifolan-LE is a purified (1→3)-β-D-glucan with single (1→6)-β-linked glucosyl side chains every third backbone chain repeat unit on average. They assigned specific proton NMR resonances to the anomeric proton of the glucosyl side chain repeat unit and to one of the methylene protons of the branchpoint glucosyl repeat unit in the backbone chain connected by a (1→6)-β-linkage. In the proton NMR spectrum, the resonances for the anomeric proton and methylene proton were of equal integrated area, which is consistent with a side chain structure containing only a single repeat unit. The proton NMR spectrum of the glucan isolated from *C. glabrata ace2* strain (**Fig. 1**) resembles the NMR spectrum of Grifolan-LE except that the integral areas of the anomeric proton and one of the methylene protons involved in the (1→6)-β-linkages in the side chain are not of equal area. In addition, the NMR spectrum (**Fig. 1**) shows 5 major resonances previously assigned to glucosyl repeat units of a (1→3)-β-D-glucan polymer chain[11]. These assignments were confirmed by COSY 2D NMR (data not shown) and are summarized in **Table 1**.

**Table 1:** Proton (top) and ^13^C (bottom) NMR chemical shifts (in ppm) for structural features characterized in the glucan isolated from *C. glabrata ace2 (HLS122)* strain.

Chemical Shift, ppm							
Proton Assignment	(1→3)-β-Linked Backbone Chain	Br	SC1	SC Internal (1→6)-β-Linked Side Chain	SC SNRT (1→6)	SC NRT (1→6)	NRT (1→3)
**H1**	4.53	4.36	4.25	4.27	4.27	4.37	4.39
**H2**	3.31	3.24	3.03	3.04	3.04	3.10	3.09
**H3**	3.48	3.39	3.19	3.19	3.19	3.23	3.22
**H4**	3.27	3.32	3.15	3.15	3.16	3.15	3.15
**H5**	3.27	3.23	3.32	3.32	3.33	3.25	3.25
**H6**	3.73	4.00	4.01	4.01	4.03	3.72	3.72
**H6′**	3.48	3.64	3.61	3.61	3.66	3.46	3.46
							
**Carbon Assignment**							
**C1**	102.70	102.43	103.10	102.96	102.96	103.66	103.48
**C2**	72.54	71.99	73.60	73.16	73.16	76.47	76.57
**C3**	85.97	87.14	76.32	76.31	76.32	75.89	75.95
**C4**	68.15	67.90	69.74	69.74	69.79	69.79	69.79
**C5**	76.08	75.88	75.13	75.23	75.22	76.48	76.48
**C6**	60.63	67.86	68.24	68.17	68.23	60.82	60.84

**Figure 2:**
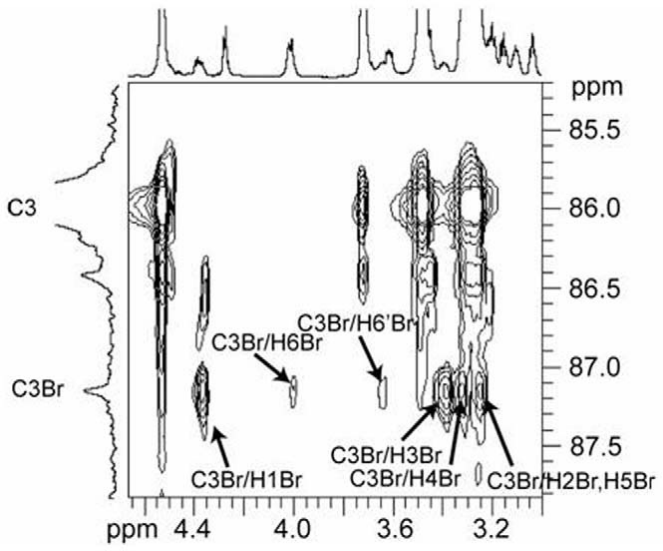
Initial identification of ^13^C and ^1^H chemical shifts for the branchpoint repeat unit of the (1→3,1→6)-β-D-glucan isolated from *C. glabrata ace2* strain. HSQC-TOCSY 2D NMR spectrum shows correlations between C3 and C3Br carbons and methylene protons in the same spin system as C3Br.

**Figure 3:**
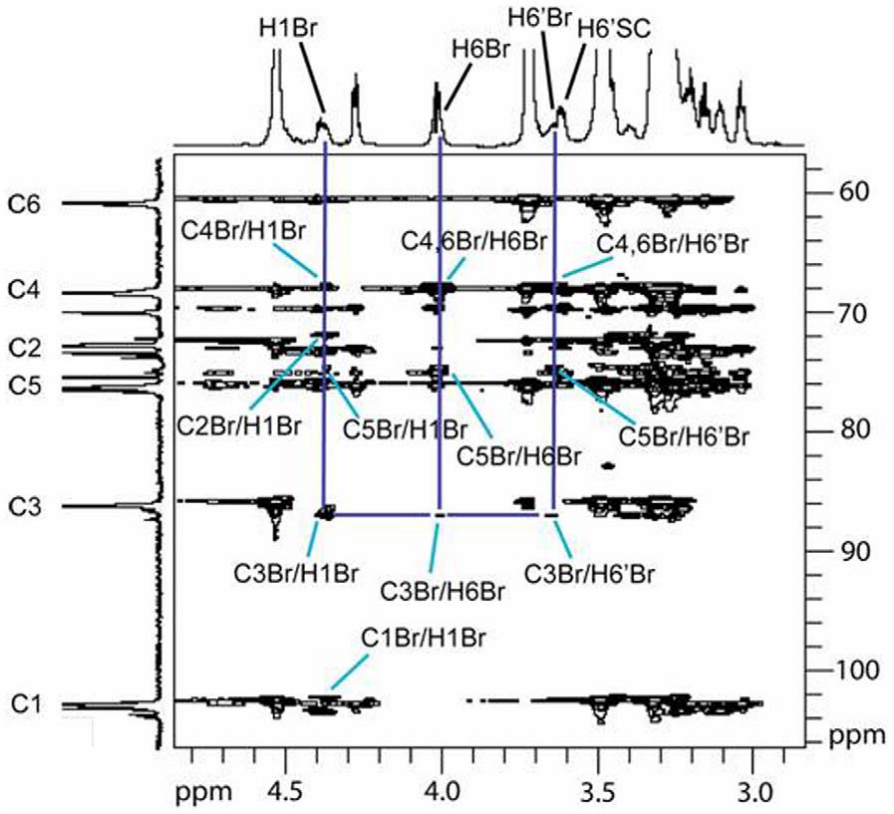
Assignment of ^13^C and ^1^H chemical shifts for the branchpoint repeat unit of the (1→3,1→6)-β-D-glucan isolated from *C. glabrata ace2* strain. HSQC-TOCSY spectrum indicates assignments and correlations for protons and carbons in the branch point repeat unit based upon correlations initially identified for C3Br (Figure 2).

**Figure 4:**
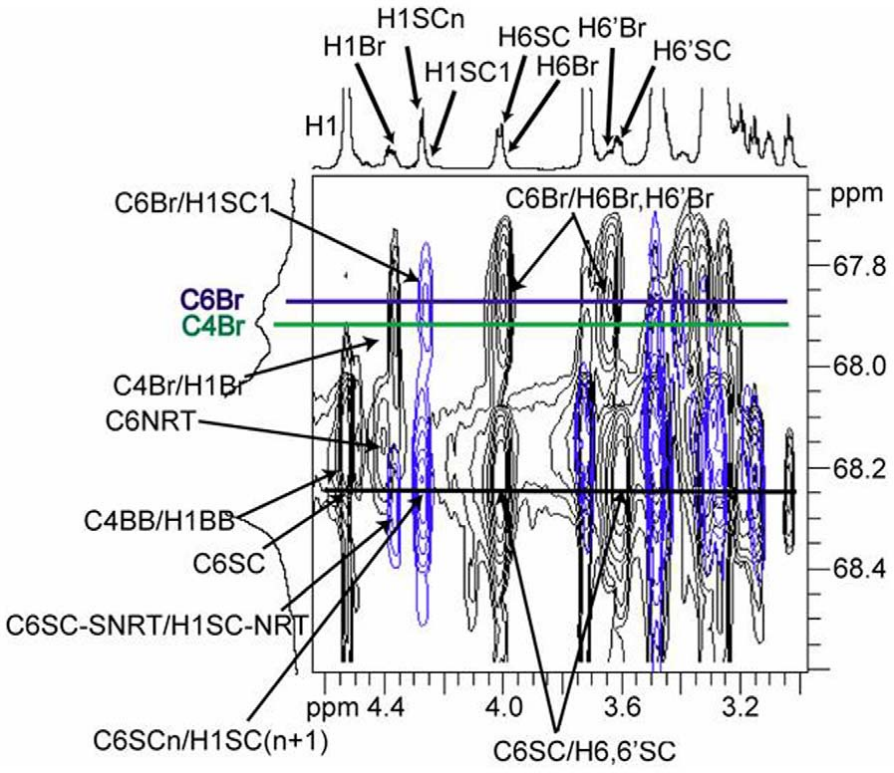
2D NMR spectra show linkage between branchpoint and the first side chain repeat unit of the (1→3,1→6)-β-D-glucan isolated from *C. glabrata ace2* strain. Overlay of the 2D HSQC-TOCSY (black) and HMBC (blue) NMR spectra expanded around the C4 spectral region shows the correlation (C6Br/H1SC1) across the glycosidic link between C6Br of the branchpoint repeat unit and the anomeric proton (H1SC1) of the first (1→6)-β-linked repeat unit in the side chain as well as correlations across other (1→6)-β-linked side chain glycosidic linkages.

**Figure 5:**
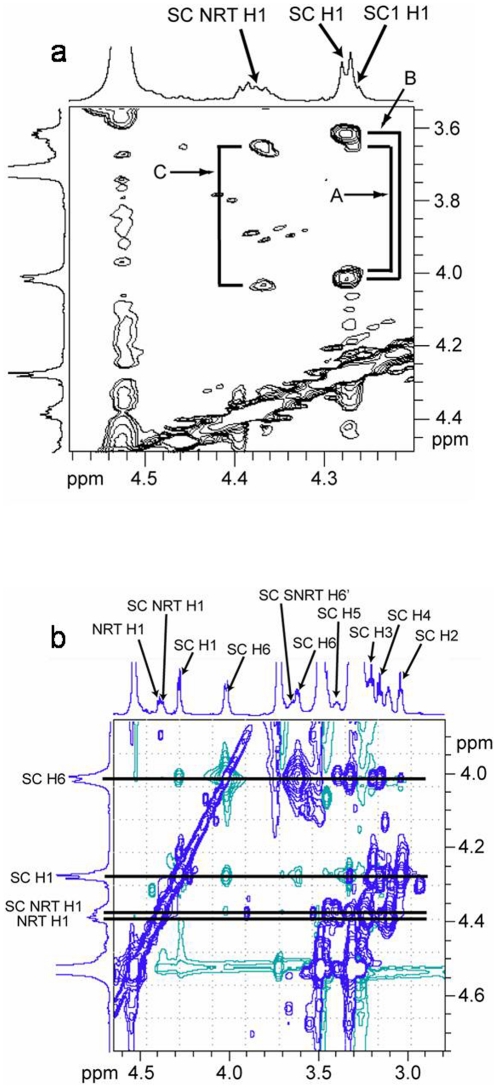
2D NMR spectra of the glycosidic linkages and non-reducing termini of the (1→3,1→6)-β-D-glucan isolated from *C. glabrata ace2* strain. (a) The three different (1→6)-β-linked glycosidic bonds from the side chain are detailed in the NOESY 2D NMR spectrum for SC1, SC Internal, and SC NRT glucosyl groups associated with H1 SC1, SC H1 and SC NRT H1. A: H6Br,H6′Br/H1SC1; B: H6SCn,H6′SCn/H1SC(n+1); C: H6SC(n-1),H6′SC(n-1)/H1SCNRT. (b) Similarity of the structures of the glycosyl group associated with SC NRT H1 and NRT H1 is indicated in the TOCSY 2D NMR spectrum.

**Figure 6:**
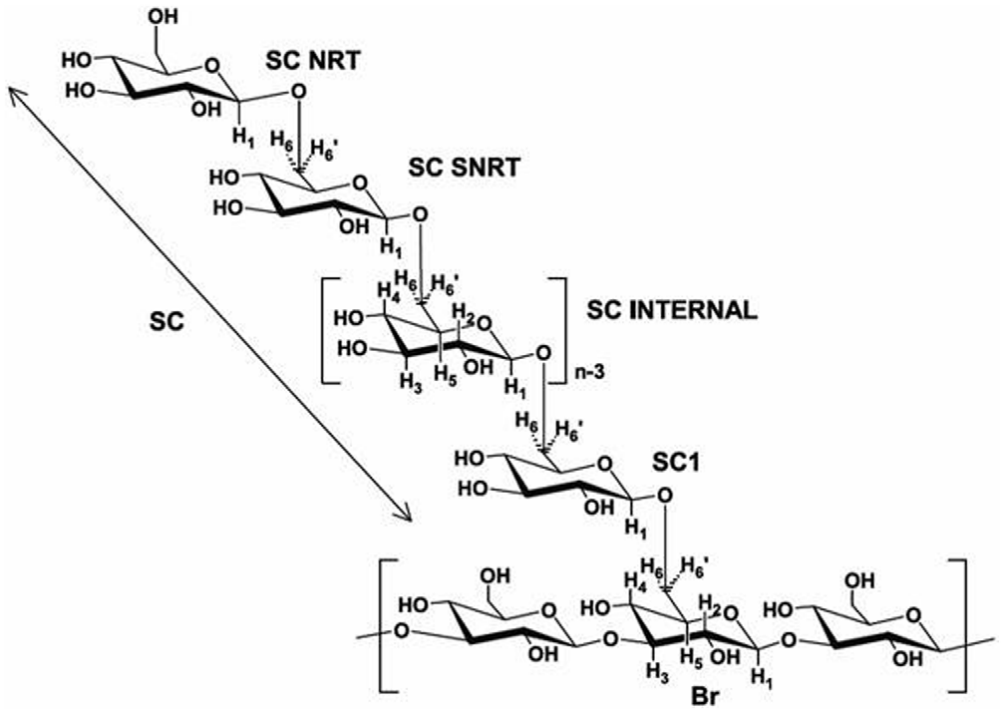
Schematic structure of the poly-(1→6)-β-D-glucan side chain containing n repeat units and attached to a segment of (1→3)-β-linked backbone chain.

The branchpoint glucosyl repeat unit (Br) in the (1→3)-β-linked polymer chain is unique in that it is the only repeat unit in either the backbone chain or the side chain that is substituted in three positions – the 1-, 3-, and 6-positions. Using the spectral region of the C3 carbon in the HSQC-TOCSY 2D NMR spectrum (**Fig. 2**), only the carbon resonating at 87.14 ppm (C3Br) exhibits long-range correlations to equivalent methylene protons at 4.00 (C3Br/H6Br) and 3.64 (C3Br/H6′Br) ppm. Other protons correlated to the C3Br resonance include those assignable to the branchpoint repeat unit (**Table 1**) as follows: H1 at 4.36 ppm (C3Br/H1Br), H2 at 3.24 ppm (C3Br/H2Br), H3 at 3.40 ppm (C3Br/H3Br), H4 at 3.32 ppm (C3Br/H4Br), and H5 at 3.23 ppm (C3Br/H5Br). These ^1^H and ^13^C NMR resonance assignments are based upon results from HSQC-TOCSY and HSQC 2D NMR experiments. The HSQC-TOCSY spectrum provides information about protons that are scalar coupled within the same repeat unit, or spin system, with one of the protons scalar coupled to a specific carbon. The strength of these correlations is dependent upon the distance of the individual protons from the specific carbon atom in the spin system and the relaxation times of the nuclei involved. Since the TOCSY spin lock time is short (60 ms), only 4 protons correlate to each carbon around the glucosyl residue in this study. Therefore, the correlation to H1Br observed from C3Br (C3Br/H1Br; 87.14 ppm/4.36 ppm) in the HSQC-TOCSY 2D NMR spectrum is important for establishing C1Br through C4Br in the branchpoint repeat unit while the correlation to H6Br/H6′Br (4.00 ppm/3.64 ppm) observed from C3Br is important for establishing C3Br through C6Br in the branchpoint repeat unit (**Fig. 3**). The correlation from the methylene carbon of the branchpoint repeat unit (C6Br) and the anomeric proton (H1) of the first side chain repeat unit (SC1) across the (1→6)-β-linked glycosidic bond is established in the HMBC 2D NMR spectrum (C6Br/H1SC1, **Fig. 4**; 67.86 ppm/4.25 ppm). The anomeric proton of SC1 correlates with H2 in the COSY 2D NMR spectrum (data not shown), and then H2 correlates with H3 and the remainder of the glycosyl protons H4 through H6/H6′ in the TOCSY 2D NMR spectrum (**Fig. 5b**). Resonances H3 through H6/H6′ of SC1 are coincident with similar resonances in internal side chain repeat units (SC INTERNAL) from the COSY (data not shown), NOESY (**Fig. 5a**), and TOCSY (**Fig. 5b**) 2D NMR spectra. Individual resonances from SC INTERNAL each exhibit the same chemical shifts (**Table 1**). Three types of (1→6)-β-linkages across glycosidic bonds are evident in the NOESY 2D NMR spectrum (**Fig. 5a**). The (1→6)-β-linked glycosidic bonds for the SC1 (H6,6′Br/H1SC1; 4.00, 3.64 ppm/4.25 ppm) and SC INTERNAL (H6,6′SCn/H1SC(n+1); 4.01, 3.61 ppm/4.27 ppm) have been identified. The remaining (1→6)-β-linkage exhibits H6,H6′ correlations to an anomeric proton with chemical shifts typical of a (1→6)-β-linkage. The anomeric proton in this linkage has a chemical shift characteristic of the anomeric proton in the non-reducing terminus (NRT H1) of the (1→3)-linked polymer backbone. TOCSY spectra of the NRT glycosyl repeat units (SC NRT H1 and NRT H1 in **Fig. 5b**) are characteristic of mono-substituted glucosyl repeat units. Therefore this third (1→6)-β-linkage is assigned to the glycosidic linkage between the methylene protons of the next to the last (second to the last) glycosyl repeat unit (SC SNRT H6,H6′; 4.03, 3.66 ppm) and the anomeric proton of the non-reducing terminus of the (1→6)-β-linked side chain (SC NRT H1; 4.37 ppm). These data complete the assignment of all repeat units and glycosidic linkages in the (1→6)-β-linked side chain of this glucan isolated from *C. glabrata* and support the side chain structure containing multiple (1→6)-β-D-glucosyl repeat units (**Fig. 6** **)**.

In addition, the anomeric proton for the α-conformer of the reducing terminus (α-RT) of the polymer backbone is assigned to 4.99 ppm while the chemical shift of the β-RT anomeric proton is overlapped by resonances from the anomeric protons of Br, SC NRT and NRT. Anomeric proton resonances for α- and β-glucose are observed at 4.93 and 4.30 ppm, respectively. Anomeric protons for the α- and β-conformers of SRT are observed at 4.46 and 4.49 ppm, respectively. These anomeric proton resonance assignments are in agreement with previous assignments by Kim and coworkers [12] and in general agreement with assignments by Størseth and coworkers [7].

### Calculation of the average side chain length and branching frequency

To determine the average chain length and its average branching frequency for side chains containing multiple (1→6)-β-D-glucosyl repeat units, the integrated areas of the resonances assigned to H1 of SC NRT, H1SC, and H6SC of the side chain glycosidic bond, and H1 of the (1→3)-β-linked repeat units in the polymer backbone were compared. Interestingly, the integrated areas of the resonances assigned to H1SC (4.27 ppm) and H6SC (4.01 ppm) of the glycosidic linkages in the (1→6)-β-linked side chain are not equal in the glucan isolated from *C. glabrata* (**inset in** **Fig. 1**). The integrated area of the H6SC multiplet resonance is larger than the area of the H1SC multiplet resonance. Integration of these two resonances gives an area ratio of 0.787∶1 for resonances H1SC and H6SC, respectively, not the 1∶1 ratio reported for Grifolan-LE with the single (1→6)-β-linked glucosyl repeat unit in the side chain [8]. The difference in areas of the two resonances represents the integral area assignable to the anomeric proton H1 in SC NRT. The ratio suggests that the average length of the side chain contains 4.7 (1→6)-β-D-linked repeat units. The ratio of the areas from the resonances assigned to H1 of SC NRT and H1 of the (1→3)-β-linked repeat units in the polymer backbone indicates that a side chain is attached to the (1→3)-β-D-linked polymer backbone on average every 21 repeat units.

### Impact of the glucan isolation scheme on the average side chain structure

Several strains of *C. glabrata* isolated under different conditions have been examined by proton NMR. Isolation methods 1A and 1B represent duplicate glucan extractions using 1 N phosphoric acid while method 2 represents glucan extracted with 2 N phosphoric acid. All other parameters of the extraction were held constant. This was done because we have previously shown that by decreasing the concentration and/or strength of the protic acid it is possible to preserve more of the native structure of the beta-glucan [26]. At the higher acid concentration, the (1→6)-β-linked side chains are 2 repeat units in length on average while the branching frequency differs for the 4 strains examined (**Table 2**). The strains, other than wild type, were chosen due to their putative role in mannan synthesis as well as potential in altering the wall structure, we were curious as to whether there were any changes in the glucan of these strains, since compensatory changes could occur with other carbohydrate components in these mutants. For wild-type and *anp1* strains, the branching frequency is about one-third larger than for the *mnn2* and *mnn11* strains. At the lower acid concentration, the average side chain length is about twice the length of that from the higher acid concentration extraction and the branching frequency is constant with side chain branches on average every twenty repeat units in the polymer backbone. Thus, the strength of the protic acid used to isolate the glucans significantly impacts the length of the side chain, *i.e.* lower acid concentration results in great preservation of the side chain architecture.

**Table 2:** Comparison of average side chain lengths, SCn, and branching frequencies, Br Freq, for several strains of *C. glabrata* isolated by 2 different methods.

Isolation Method	Strain	SCn	Br Freq
1A	Wild-type	4.0	22.2
1A	*ace2*	4.2	20.3
1A	*mnn11*	4.1	21.8
1B	Wild-type	4.4	19.7
1B	*ace2*	4.7	20.7
1B	*mnn11*	4.1	21.4
2	Wild-type	2.5	40.7
2	*anp1*	1.8	43.0
2	*mnn2*	2.0	30.8
2	*mnn11*	2.1	30.5

### Molecular modeling of the glucan side chain

**Figure 7:**
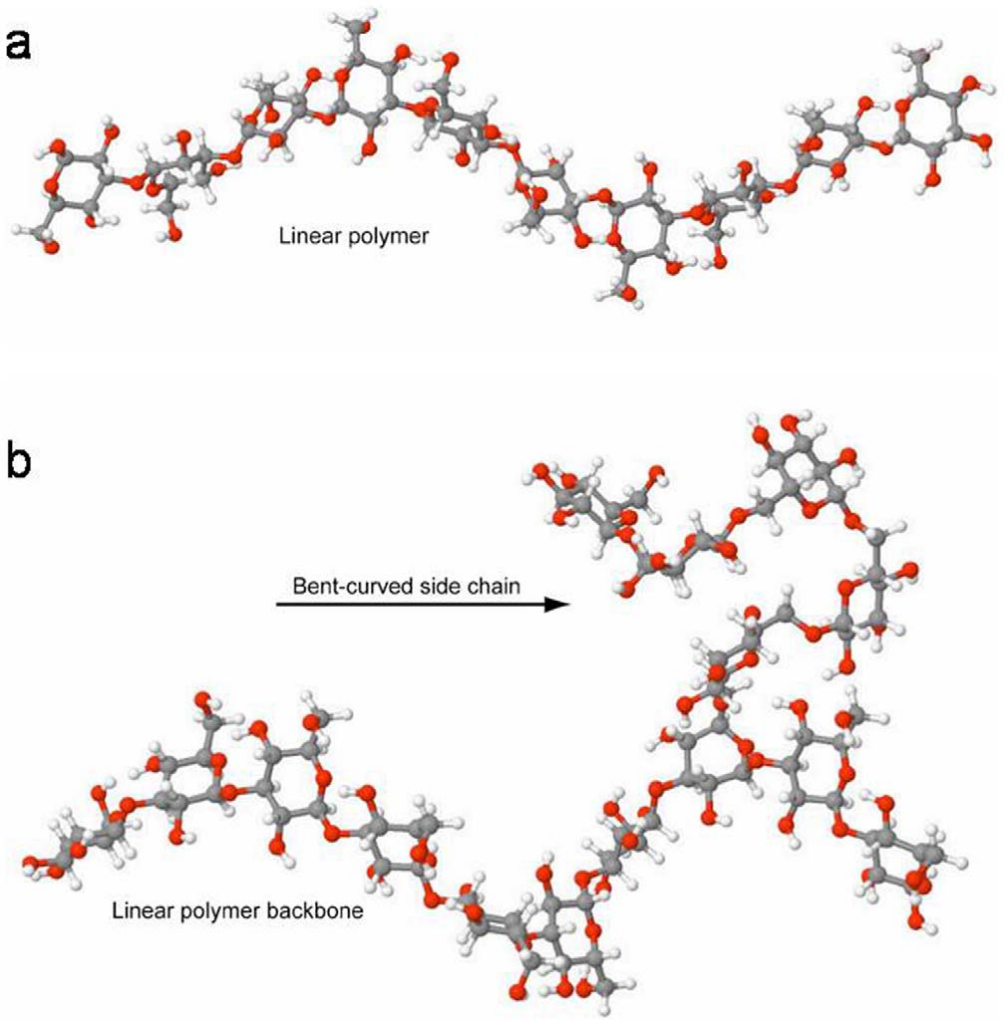
Molecular modeling suggests one possible conformation that (1→6)-β-linked glucan side chains may exhibit, that is, a hook-like, bent structure. Structure A (top) is a linear polymer containing ten (1→3)-β-linked repeat units in the polymer chain. The linear (1→3)-β-linked glucan backbone structure assumes an open helical conformation. Structure B (bottom) is the same linear structure except a side chain branches from the third repeat unit. The side chain contains five (1→6)-β-linked repeat units. The curvature and hook-like structure of the side chain is evident in this model where the structure has been rotated slightly to optimize visualization of the curved side chain. The structures are rendered using JMOL [39].

Based upon the structural characterization of the (1→6)-β-linked side chain, we explored molecular structure by performing computational chemistry. We first used molecular modeling – molecular mechanics (MM) – on a model compound consisting of a glucan with ten (1→3)-β-linked repeat units in the polymer backbone and five (1→6)-β-linked repeat units in the side chain attached to the third repeat unit in the polymer backbone. These results were compared to similar calculations for a linear polymer containing ten (1→3)-β-linked repeat units in the polymer chain (**Fig. 7**a). It is important to mention that other researchers have also used *in silico* molecular mechanics (MM) to conduct energy minimizations to probe glucans, specifically glucan:mycotoxin and glucan:zearalenone complex interactions[13], [14]. It is also important to recognize that various researchers commonly use different computational programs and/or different approaches to probe theoretical structure. Our approach utilized a local minimum of the linear form and was subsequently used to conduct a semi-empirical Austin-Model (AM1) calculation. The resulting molecular structure was then used to conduct *ab initio* geometry optimizations at the Hartree-Fock (HF)/STO-3G basis set; an approach previously used by our group to investigate theoretical structure of a linear decasaccharide and a branched nonasaccharide [15]. Addition of the (1→6)-β-linked side chain resulted in the branchpoint repeat unit (Br) exhibiting a skewed conformation. Addition of the (1→6)-β-linked side chain, initially as a linear side chain, to a linear (1→3)-β-linked polymer backbone resulted in a calculated, lowest energy side chain structure where the repeat units near the non-reducing terminus of the (1→6)-β-linked side chain curled back onto side chain repeat units closer to the branchpoint repeat unit (**Fig.7b**). While HF/STO-3G may be considered a first approximation, our main computational goal was to generate a theoretical cartoon of the branched model glucan. To improve upon the STO-3G result, we took the geometry optimization and performed *ab initio* geometry optimizations using Gaussian 03 at the Hartree-Fock level of theory using Pople basis set 6-31G(d)[16]–[18]. This basis set was designed to obtain structures that are comparable to those obtained from experimental procedures and consistently used at the Hartee-Fock level, in the gas-phase, to obtain new geometrical parameters commonly used to conduct AMBER calculations. The data obtained at 6–31G(d) were similar to the predicted structure by STO-3G (data not shown) and thereby does not alter the general non-covalent hooking interaction hypothesis.

## Discussion

Previous work by Manners and coworkers [5] proposed an often-repeated side chain structure containing a single (1→6)-β-linked glucosyl repeat unit connecting a chain of (1→3)-β-linked glucosyl repeat units to the polymer backbone. While the branchpoint repeat unit would have a 2D NMR spectral signature similar to that observed for our glucan isolate, the remainder of the side chain structure would not present a similar 2D NMR signature due to the presence of the (1→3)-β-linkages. In this current study we have presented the first, to our knowledge, detailed structural characterization of the (1→6)-β-linked side chain in (1→3,1→6)-β-D-glucan isolated from the fungal cell wall of *C. glabrata*. This detailed structural analysis will serve as a model for the global structural characterization of the glucan in terms of average (1→6)-β-linked side chain length and branching frequency along the (1→3)-β-linked polymer backbone. We have also observed that the methodology used to extract the glucan can impact the structure of the isolated glucan. In addition, molecular modeling based upon these NMR results provides new insight into how (1→3,1→6)-β-D-glucans may be incorporated into the carbohydrate architecture of the fungal cell wall.

We have observed that the H1SC and H6SC resonances from the (1→6)-β-linkage also exhibit several inflection points (**inset in** **Fig. 1**). Størseth and coworkers [7] suggest that these multiple inflection points reflect multiple chain lengths of the (1→6)-linked side chain. Two other studies showed that (1→6)-β-D-glucan polymers isolated from *Actinobacillus suis* and *Malassezia sympodialis* exhibits multiple conformations about the (1→6)-β-linked glycosidic bond resulting in additional resonances in the proton NMR spectrum of the isolate [19], [20]. Therefore, it is reasonable to consider that, while the multiple inflection points in the H1SC and H6SC resonances may result from different side chain lengths, they may also result from different conformations observed for the (1→6)-β-linked glycosidic bond, thus providing additional support for the presence of multiple (1→6)-β-linked glucosyl repeat units in the average side chain.

### Differences in acid concentration affect side chain length

Isolation of glucan from several strains of *C. glabrata* using two different methods illustrates the role isolation protocol plays relative to the final structure of the isolated glucan. At the lower phosphoric acid concentration, the average side chain length is about twice the length of that from the higher phosphoric acid concentration while the average distance between branches along the polymer backbone is less (**Table 2**). The higher acid concentration results in reduced side chain length as well as side chain content on average compared to the lower acid concentration. The resulting glucan structures from these two different isolation methods clearly demonstrate the importance of consistency in the isolation scheme. The structure of the isolated glucan can be dramatically impacted by differences in isolation protocols, which can impact ligand-receptor binding studies as well as action of the glucan in a biological system. Thus, our data indicate that isolation conditions which are less harsh, *i.e.* lower acid concentration, result in preservation of the glucan side chain structure. We speculate that milder isolation conditions result in side chain structures that are most closely representative of the side chains found in the fungal cell wall.

The nature of the side chains, i.e., their structure and branching frequency, and how they impact ligand-receptor interactions are important topics relative to understanding how pathogenic and non-pathogenic fungi interact with their environment, including the innate immune system. From a human health standpoint, glucans are critical to the structure and function of the fungal cell wall. They are the primary pathogen associated molecular patterns (PAMPs) recognized by the innate immune system [15], [21], and provide a drug development target for glucan synthase inhibitors, the newest anti-fungal drugs.

### A structural feature leads to new insights about glucan organization

**Figure 8:**
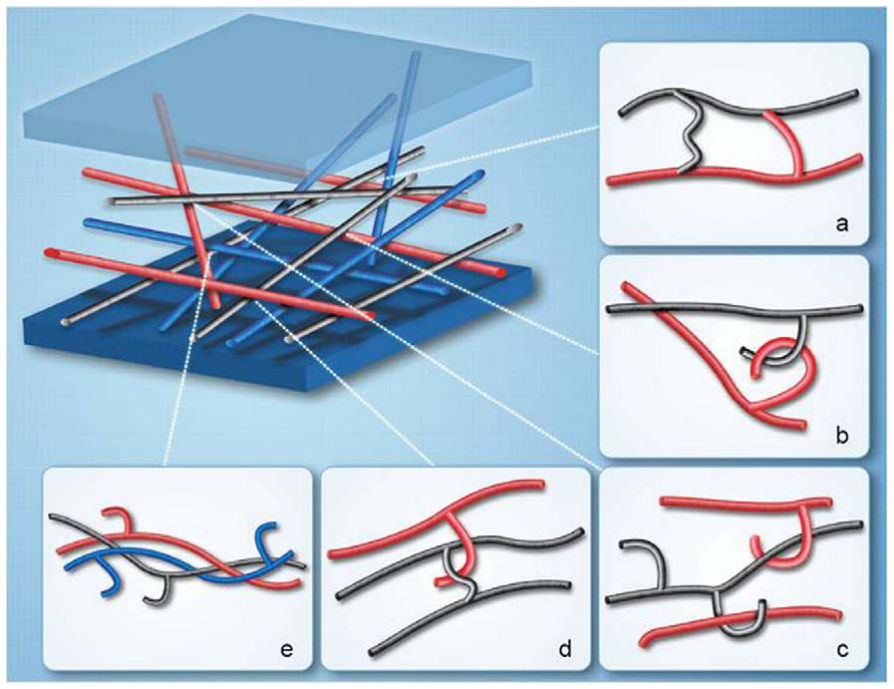
Conceptual model of the role of (1→6)-β-linked glucosyl side chains in the fungal cell wall structure. Possible arrangements of (1→6)-β-D-glucan branches within the glucan matrix include (a) direct covalent cross-linking between two (1→3)-β-linked polymers, or (b) non-covalent interactions by “hooking” across (1→6)-β-linked side chains, or (c) across (1→3)-β-linked polymers, or (d) “catching” a covalent (1→6)-β-linked cross-linking branch. The triple helix arrangement (e) with associated (1→6)-β-linked side chains may serve as points for attachment to chitin, mannan, mannoprotein, GPI protein anchor or other possible molecules.

Adams and coworkers [15] have discussed the importance of these side chain structures relative to interaction of (1→3,1→6)-β-D-glucan PAMPs with Dectin-1, the primary pattern recognition receptor (PRR) for glucans [21]. To better understand how these glucans with longer side chains may interact with Dectin-1 as well as neighboring polymer chains, theoretical optimized molecular geometries were investigated for the model compound containing five (1→6)-β-linked repeat units in the side chain (**Fig. 7**). This side chain structural proposal is interesting in that it may offer insight into how these side chains aid in not only the rigidity and structure, but also the plasticity, of the fungal cell wall [22]. At present, it is not clear whether glucan polymer strands within the fungal cell wall are interlinked, and if they are, it is not clear how this is accomplished. The side chains have non-reducing termini, which would not allow the formation of covalent linkages between glucan polymer strands. However, the curled side chain may provide a mechanism for interaction of the side chain with other glucan chains by wrapping around other polymer chains, thus not requiring covalent bonding between the side chain repeat units and other chains in the polymer. We propose that the curled side chain structures may connect neighboring glucan polymer chains in a manner that maximizes wall strength (**Fig. 8a**), while also allowing flexibility, or plasticity, in the fungal cell wall. In addition to a direct covalent interaction between strands of polymer, there are several possible interactions through hydrogen bonding, through which the side chains can interact. First there could be a direct hooking of two (1→6)-side chains from two individual (1→3)-linked polymers (**Fig. 8b**) allowing for a mesh like structure to form; this interaction with the hooking of a (1→6)-branch around a (1→3)-linked polymer (**Fig. 8c**) would give both strength and flexibility allowing for “slippage” of the (1→6) side chain down the length of the (1→3) polymer. Another arrangement could be the catching of a (1→6) branch that is cross-linked to a second branch with a hooked branch on a third polymer (**Fig. 8d**) forming a “catch” structure similar to that seen in **Fig. 8b**. As much of the (1→3) glucan is arranged in a triple helix (**Fig. 8e**) the presence of (1→6) side chains may play a lesser role in the integrity of the glucan matrix and may serve as point of attachment for other molecules such as mannan, mannoprotein, chitin or some other molecules not yet identified in the cell wall. Additional investigation will be required to fully elucidate the role of (1→6) side chains in fungal cell wall architecture. However, what we have proposed provides a potential explanation for the rigidity, flexibility and interconnections that the fungal cell wall exhibits.

## Materials and Methods

### Strains and growth conditions

*C. glabrata* strain ATCC2001 was obtained from the American Type Culture Collection. *C. glabrata ace2* (HLS122) strain has been described previously [23]. *C. glabrata mnn2*, *mnn11,* and *anp2* strains were constructed by gene replacement [24]. Strains were routinely cultured on YPD (1% yeast extract, 2% peptone, 2% dextrose, 2% agar) at 30°C.

### Glucan isolation

Strains were cultured in 1L of YPD at 30°C for 18 hr with agitation. Cells were harvested and then lyophilized. The lyophilized cells were extracted using modifications of the methods of Williams, Mueller and coworkers [25], [26]. Specifically, in method 1 the phosphoric acid concentration was 1 N, while in method 2 the phosphoric acid concentration was 2 N. Decreasing the phosphoric acid concentration reduces polymer degradation during isolation [26]. It is important to note, that this extraction method does not result in phosphorylation of the glucan. All other aspects of the method were as previously reported by our group [25], [26].

### NMR

Proton and ^13^C NMR spectra were collected on a Bruker Avance II 800 NMR spectrometer using a 5 mm TCI inverse cryoprobe operating at 343°K. For initial NMR spectra, ∼25 mg of glucan was solvated in 950 µL DMSO-d_6_ (Sigma-Aldrich, “100”, p/n 156914, CAS 2206-27-1) with 40 µL trifluoroacetic acid-d (Cambridge Isotope Laboratories, 99.8+% deuterated, p/n DLM-46, CAS 599-00-8) to shift the exchangeable proton resonances downfield. Approximately 550 µL of the sample was placed into a 5 mm NMR tube. Trifluoroacetic acid was added just prior to NMR analysis, in order to reduce the exposure time during which depolymerization could occur. For ^13^C NMR spectra, a second sample was prepared using ∼15 mg of glucan dissolved in 600 µL DMSO-d_6_ with no added trifluoroacetic acid and transferred to a 5 mm NMR tube. Proton and ^13^C 1D and COSY [27], [28], NOESY [29], [30], TOCSY [31], HSQC [32]–[34], HSQC-TOCSY [35], [36], HSQC-NOESY [35], and HMBC [37] 2D NMR spectra were obtained in this study. For 1D NMR experiments, chemical shift referencing used absolute referencing based on the ^2^H DMSO-d_6_ lock signal and ^1^H and ^13^C gyromagnetic ratios. For 2D NMR experiments, chemical shift referencing was accomplished relative to the anomeric resonance of the (1→3)-β-linked backbone repeat unit at 4.53 ppm for ^1^H and 102.70 for ^13^C relative to DMSO-d_6_ residual protons as referenced above. We performed all ^1^H homonuclear 2D NMR spectra on one sample and all ^13^C-{^1^H} heteronuclear 2D NMR spectra on a second solution of the same sample. 1D NMR spectra were collected and processed as follows: for ^1^H NMR, 64 90° scans with 8 dummy scans, acquisition of 65,536 real points zero-filled once and Fourier transformed to 65,536 complex points, 10.5 ppm sweep width, centered at 3.85 ppm with a total acquisition time of 3.9 s, exponential apodization with 0.3 Hz broadening, and 3 s pulse delay for a total interpulse period of 6.9 s; for ^13^C NMR, 16,384 30° scans with 8 dummy scans, acquisition of 65,536 real points zero-filled once and Fourier transformed to 65,536 complex points, 65 ppm sweep width, centered at 80 ppm with a total acquisition time of 2.5 s, exponential apodization with 3 Hz broadening, and 2 s pulse delay for a total interpulse period of 4.5 s. 2D NMR spectra were collected and processed as follows: for homonuclear NMR experiments, 512×256 point matrix was zero-filled to 2048×1024 points, 32 scans per row with 16 dummy scans, 3 ppm sweep width centered at 3.85 ppm, cosine-squared apodization in both dimensions, and 2 s pulse delay; for heteronuclear NMR experiments, 512×512 point matrix was zero-filled to 1024×1024 points, 64 scans per row with an initial 64 dummy scans, 3 ppm sweep width centered at 3.85 ppm for the f2 dimension and 80 ppm sweep width centered at 65 ppm for the f1 dimension, cosine-squared apodization in both dimensions, and 1.5 s pulse delay except for HMBC which was zero-filled to 4096×1024 points and sine apodized. Adiabatic ^13^C decoupling was used during the t_2_ acquisition period of the heteronuclear experiments, except for the HMBC experiment, and shaped inversion pulses were used on the ^13^C channel in inverse heteronuclear experiments. NMR spectra were processed using TOPSPIN 2.1 running on the Avance II 800 NMR and TOPSPIN 3.0.b.8 running on Windows XP Professional operating system under VMWare Fusion version 2.0.5 (VMWare, Inc., Palo Alto, CA) on a Macintosh MacBook Pro.

### Molecular modeling

Chemical structures were drawn in a linear format using CS ChemDraw Ultra® (version 6.0.1; Cambridge Soft Corporation; Cambridge, MA). The structures were then copied into CS Chem3D Ultra®. For each polysaccharide, a molecular mechanics (MM) minimization was conducted using a root-mean-square (RMS) of 0.005. Next, molecular geometries were generated in the Gaussian Z-matrix style via the CS MOPAC application. For each compound, an Austin-Model (AM1) semi-empirical calculation (closed shell, tight convergence criteria) was conducted using the Gaussian^©^ software package (Gaussian, Inc.; Carnegie, PA) [38]. *Ab initio* geometry optimizations were subsequently performed using Gaussian 03 at the Hartree-Fock level of theory using the STO-3G [16], [17] basis set. Additional a*b initio* geometry optimizations were performed using Gaussian 03 at the Hartree-Fock level of theory using the 6–31G(d) basis set and compared (data not shown) to STO-3G results[16]-[18]. For STO-3G analysis geometry optimizations using implicit solvation were performed with the Onsager method [18] using the dielectric constant of water (78.39) and the solute radius; calculated with the more accurate molecular volume computation (Volume_Tight). These calculations were performed with a Microway AMD (Microway Technology, Plymouth, MA) dual 64 bit 2.0 GHz central processing unit (CPU) with 4GB RAM running Fedora Core 3 and an Aspen Systems 30 CPU cluster (Wheatridge, CO). Each blade contained two 32-bit Xeon 3.2 GHz CPUs with 4GB RAM running Red Hat Linux.
